# Supplementary material for: Development of Radiofluorinated Nicotinamide/Picolinamide Derivatives as Diagnostic Probes for the Detection of Melanoma
Source: Int J Mol Sci. 2021 Jun 16;22(12):6432. doi: 10.3390/ijms22126432 (PMC8234188; doi:10.3390/ijms22126432)
Supplement: Supplementary file 1 [file ijms-22-06432-s001.zip › ijms-1250720-supplementary.pdf]

Supplementary Materials:

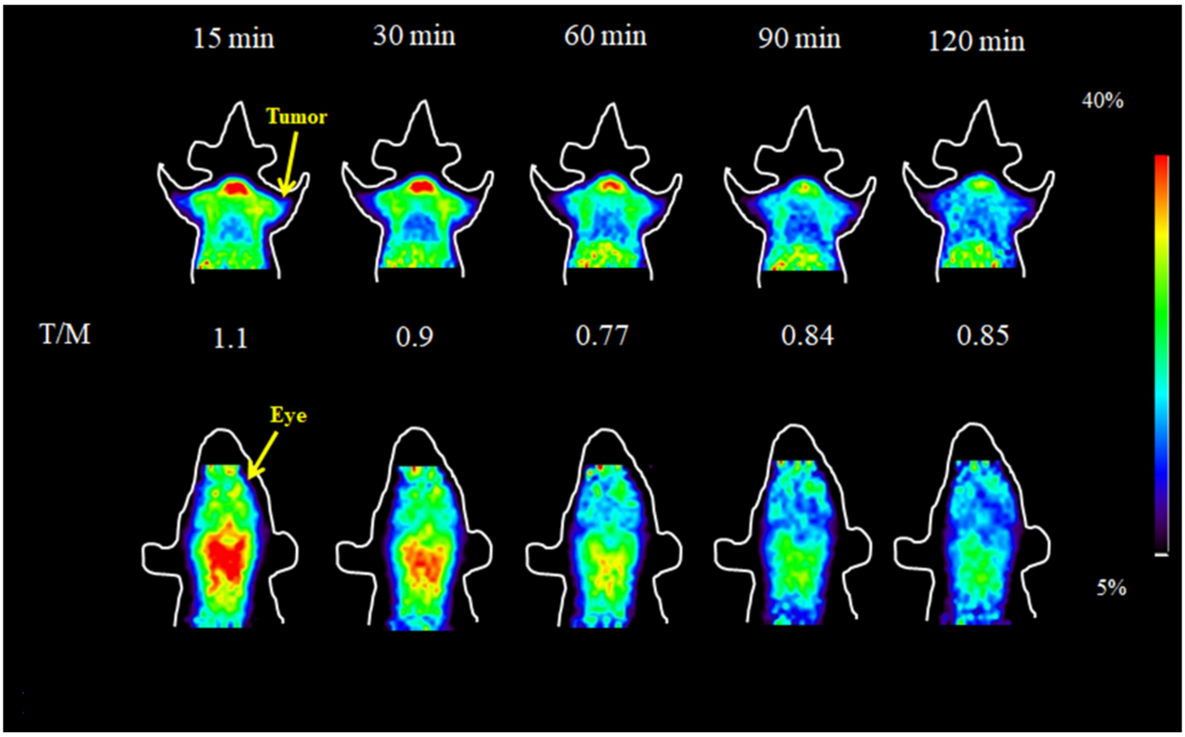

Figure S1. Representaive microPET images of A375 tumor-bearing nude mice administered with ^{18}F-FPABZA. The image slices covered the tumor (top) and eyes (bottom). T/M: tumor-to-muscle ratio.
